# A COVID-19 Diagnosis Like an Avalanche Triggers a Series of Adverse Events but Saves a Life in the End

**DOI:** 10.3390/healthcare11131847

**Published:** 2023-06-26

**Authors:** Mateusz Iwański, Aldona Sokołowska, Piotr Wańczura, Justyna Filipowska, Katarzyna Styczkiewicz

**Affiliations:** 1 College for Medical Sciences, University of Rzeszow, 35-315 Rzeszow, Poland; 2 Department of Cardiology, The Ministry of Internal Affairs and Administration Hospital, 35-111 Rzeszow, Poland; 3 Subcarpathian Oncological Center, University Clinical Hospital of Frederic Chopin, 35-055 Rzeszow, Poland

**Keywords:** COVID-19, cancer, thrombotic complications, oncologic therapy

## Abstract

Patients diagnosed with cancer are less frequently covered by preventive measures for cardiovascular diseases. The frequent co-occurrence of these diseases makes it necessary to apply parallel diagnostics and cardiological treatment with anti-cancer therapy. Frequently. multidisciplinary team discussions are needed. Case report: We present a case of a 73-year-old former smoker with hyperlipidemia, type 2 diabetes, and arterial hypertension, after a partial right nephrectomy in 2005 due to kidney cancer, diagnosed with SARS-CoV-2 infection in April 2022. Subsequent chest imaging showed a 20 mm focal lesion in the left lung further classified as a small-cell neuroendocrine carcinoma. Unexpectedly, the patient was hospitalized due to ST-segment elevation inferior left ventricular (LV) myocardial infarction. It was treated successfully with percutaneous coronary angioplasty (PCI) of the circumflex and first marginal artery with drug-eluting stent (DES) implantation. One day later, PCI of the left anterior artery was performed with two DES implantation; however, heart failure (HF) with a reduced left ventricle ejection fraction of 30% was diagnosed. One month later, the patient required hospitalization again due to HF decompensation, and cardiological treatment was optimized with flozin in addition to standard HF therapy. Subsequently, after cardiological approval the patient qualified for chemotherapy with the cisplatin–etoposide regimen. Therapy was continued for 6 months without HF decompensation and significant deterioration in renal function. After that, the patient underwent radical radiotherapy. Follow-up chest computed tomography scans showed regression of the neoplastic lesion. Conclusions: The coincidence of newly recognized cancer and inflammatory disease might contribute to and provoke serious cardiological events. To reduce the risk of cardiovascular complications, periodic cardiological surveillance and optimal pharmacotherapy are required.

## 1. Introduction

The majority of deaths worldwide are caused by cardiovascular diseases (CVDs) and cancer [1,2]. Over the last few decades, it has been discovered that both CVDs and cancer are more common in individuals with traditional cardiovascular risk factors accumulation. Several risk factors that are common to CVDs and cancer make preventative measures extremely effective in reducing disease incidence [3,4]. In recent years, the bidirectional association between myocardial infarction (MI) and cancer has been established. One prospective cohort study described the future risk of incident cancer in patients diagnosed with MI. The study (n = 28,763) included participants without a previous history of MI or cancer and who had a follow-up period of more than 15 years. There were 1747 participants with MI, 146 of whom developed cancer afterwards. The patients with MI had an increased risk of 46% of developing cancer compared with those without MI [5]. On the other hand, a significant increase in arterial thromboembolism risk is associated with incident cancer [6]. Reasons for thrombotic increased risk include cancer patients’ immobility, invasive procedures, and alterations in coagulation and platelet and endothelial function related to both the cancer’s presence and cancer systemic treatment. In Navi B. et al.’s study [6], lung cancer had the greatest excess risk of thromboembolism, which also correlated with cancer stage. This study also revealed that in newly diagnosed cancer patients, the 6-month cumulative incidence of myocardial infarction was markedly higher than that of matched control patients (HR: 2.9, 95% CI: 2.8 to 3.1; *p* < 0.001) [6]. The authors even suggested that patients with newly diagnosed malignant cancer, particularly those with advanced disease, may be considered as candidates for antithrombotic and statin treatment for the primary prevention of CVDs [6]. However, given that cancer patients are also prone to bleeding due to frequent coagulopathy and thrombocytopenia, these questions need to be answered in the future, in carefully designed clinical trials.

During cancer treatment, patients may suffer cardiovascular side effects or worsening of an underlying cardiovascular condition. More and more patients are living longer due to improved oncology treatment. Cancer chemotherapy can be associated with coronary disease, severe hypertension, and thromboembolic ischemia, as well as cardiac arrhythmias. It has been established that cancer treatment can exert late effects many years after treatment. Myocardial disease, myocardial fibrosis, cardiomyopathy, coronary artery disease, and valve disease can all be induced by chest radiation, early or late [7]. Oncology patients suffering from cardiotoxicity, specifically left ventricular dysfunction, are at high risk of morbidity and mortality in the long term. Currently, there is an increase in the use of biomarkers to detect cardiotoxicity at an early stage that can be reversed [8]. Patients with cancer and cancer survivors are at an increased risk of incident heart failure (HF) and other cardiovascular events. In Paterson et al.’s study [9], a total of 224,016 participants with new cancer diagnoses were identified, as well as 73,360 cardiovascular deaths and 470,481 nonfatal cardiovascular events. Cardiovascular risk was highest for patients with genitourinary, gastrointestinal, thoracic, nervous system, and hematologic malignancies [9]. According to another retrospective cohort study including 27,195,088 individuals, participants with CVDs had an increased risk (12%) of incident cancer compared with those without CVDs. The risk was most pronounced among individuals with atherosclerotic CVD, who had a higher risk of cancer than those without CVDs. The results showed that atherosclerotic CVD was particularly linked with several malignancies, including lung, bladder, liver, colon, and hematologic cancers [10].

## 2. Case Presentation Section

A 73-year-old male former smoker with hyperlipidaemia, type 2 diabetes, and hypertension after partial right nephrectomy in 2005 due to the kidney cancer was diagnosed with SARS-CoV-2 infection in April 2022. Due to the symptomatic course of coronavirus disease (COVID-19), a chest X-ray was performed which revealed pathological changes in the left lung. Therefore, subsequent angio-chest tomography (CT) was performed, which excluded pulmonary embolism but confirmed a 20 mm-diameter subpleural focal lesion in four segments of the left lung. According to the classification of tumours (T), nodes (N), and metastases (M), this was diagnosed as cT1bN1M0, grade IIB, a small-cell neuroendocrine carcinoma.

One month later, the patient was admitted to the hemodynamic lab with inferior wall ST-segment elevation myocardial infarction. Upon admission, significant elevation in troponin T and N-terminal prohormone of b-type natriuretic peptide (NT-proBNP) was noted as well as poor glycemic control (Table 1).

**Figure 1:**
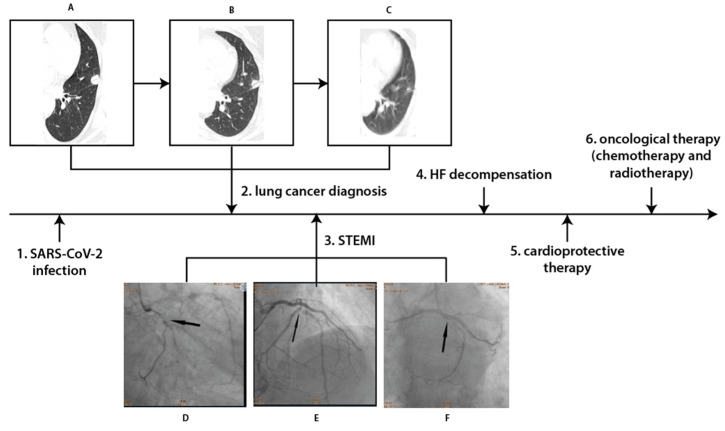
The flowchart of adverse events following SARS-CoV-2 infection. Chest CT (**A**) before treatment; small tumor diameter 20 mm located in the peripheral part of the left lung (**B**) after 3 months of therapy; and tumor mass reduction (diameter 15 mm) (**C**) after 6 months of therapy: further tumor mass reduction (diameter 8 mm). Coronary angiography (**D**) critical stenosis in the Cx culprit lesion (**E**) critical stenosis in the LAD; and (**F**) final result after Cx-OM bifurcation stenting. Abbreviations: CT—computed tomography, Cx—circumflex coronary artery, LAD—left anterior descending artery, OM—obtuse marginal artery, and STEMI—ST-elevation myocardial infarction.

Coronary angiography revealed multivessel coronary disease with significant stenoses in the circumflex artery (Cx), first marginal artery, and left anterior artery (LAD) (Figure 1). They were subsequently treated by percutaneous coronary intervention (PCI) with drug-eluting stent (DES) implantation in the Cx and first marginal artery on 26 May 2022. One day later, PCI of the LAD was performed with two DES implantation. HF with reduced left ventricle ejection fraction (LVEF) was diagnosed with LVEF 30% in echocardiography. The patient was discharged 4 days later on dual antiplatelet therapy (aspirin and clopidogrel), beta-blockers, angiotensin converting enzyme inhibitors, furosemide, and statins. A mineralocorticoid receptor blocker was not introduced in this case due to the low blood pressure values.

The patient was hospitalized again at the cardiology department in June 2022 due to acute HF decompensation. Upon admission, their oxygen saturation was 86%, and the following abnormalities were observed upon laboratory examination—in Table 1, in particularly, the D-dimers and NT pro BNP levels were elevated. Due to the clinical symptoms, mainly severe dyspnoea and congestion in the pulmonary circulation noted on chest X-ray, intensive diuretic treatment with intravenous furosemide was initiated, resulting in a gradual improvement in the patient’s clinical condition and the remission of dyspnoea. Upon discharge, the patient’s estimated glomerular filtration rate was 57 mL/min/1.73 m^2^. In addition to previous therapy, dapagliflozin 10 mg once daily was introduced.

One month later, the patient was admitted to the department of clinical oncology to qualify for systemic treatment. The patient was stable with no HF decompensation events in the previous month, with a still reduced LVEF of 30% in echocardiography. Cardiological treatment was maintained, and after multidisciplinary team discussion, chemotherapy was initiated based on a cisplatin–etoposide (PE) combination scheme. During hospitalization, the patient received the first course of the PE regimen without complications.

A chest CT scan performed in August 2022 revealed regression of the left lung neoplastic lesion (Figure 1). There were also no signs of metastases on CT scans of the head and abdomen. Chemotherapy was continued for 6 months with no HF decompensation events and no significant renal function worsening. Furthermore, the patient also qualified for and completed radical radiotherapy. Two months later, the patient was still stable with no HF decompensation events, no significant renal function worsening, and improvement in glycemic control (glycated hemoglobin 7.4%). In December, an improvement in their LVEF was also noticed from 30 to 40%.

## 3. Discussion

The growing avalanche of adverse events in our patient followed an interesting sequence. The diagnosis of COVID-19 with related imaging tests was decisive in the detection of lung cancer, which contributed to early diagnosis and radical oncological treatment. Since there is a positive correlation between the time of diagnosis and cancer prognosis, we might speculate that in this case, the post-COVID tests saved the patient’s life.

In our case, a strong correlation between COVID-19, cancer, and myocardial infarction needs to be addressed. CVDs and cancer share many overlapping common cardiovascular risk factors and mechanisms, including inflammatory processes, impaired endothelium function, and altered platelet function. On the one side, it causes these two diseases to frequently co-exist; on the other, patients are more prone to thrombotic events [11]. Cancer patients also have an additionally increased risk of thrombosis and adverse CVD events due to cancer-specific and systemic-therapy-related risk factors. COVID-19 additionally increases the risk of thrombotic complications, which triggered the cascade of adverse events in our case [12]. In addition to this, CVDs and cancer may influence the progression of each other. It has been documented that the presence of MI accelerates breast cancer growth and increases cancer-related mortality. Diabetes, which was present in our patient and is a well-known classical risk factor for CVD, has been shown to be associated with an increased risk of malignancy. The same situation is true for older age in our patient. On the one side, it is a significant risk factor for CVDs; on the other, age contributes to an increased number of somatic mutations, leading to carcinogenesis processes [12]. In patients with already diagnosed cancer, the background presence of pre-existing risk factors together with the treatment of malignancy may also have adverse effects on the development and progression of CVDs. A number of cytotoxic chemotherapy regimens have been known to cause cardiotoxicity and vascular toxicity, which may further increase atherosclerotic risk. Cisplatin and other platinum-based chemotherapeutic regimens have been associated with an increased risk of MI via endothelial damage and plaque erosion and in some cases may induce HF. Moreover, cisplatin requires the administration of high volumes of intravenous fluids to avoid renal toxicity, and patients with pre-existing CVDs and HF (like in our presented case) are at particularly increased risk of developing symptomatic HF due to fluid overload [7,12].

Radiotherapy, which is used in the treatment of various types of cancers, has been shown to increase the long-term risk of CVDs. The incidence and progression of radiation-related cardiovascular complications depend on the dose, the location involved, especially the heart field, concomitant cancer therapies, and patient characteristics, such as pre-existing CVDs and age. Radiation accelerates pre-existing atherosclerosis leading to increased MI risk within 10 years of treatment [7,12]. Our patient underwent cisplatin-based systemic therapy and radiation already after the MI event and HF development. For these reasons, the initial decision about starting such therapy was particularly difficult for the team. It also needs to be underlined that the patient is still at significant CVD risk in the future, requiring strict secondary preventive strategies and multidisciplinary care, including oncologists, cardiologists, and possibly nephrologists in long-term surveillance.

The severity of COVID-19 and mortality rates are higher when there is an underlying CVD. Depending on the severity of the condition, COVID-19 may result in primary (myocarditis, MI, and arrhythmias) or secondary cardiac complications (usually due to systemic inflammatory syndrome resulting in acute myocardial injury or congestive heart failure). In some cases, severe circulatory failure may develop [13].

Several mechanisms of MI in cancer patients have been proposed [14,15,16]. The metastases of lung cancer to the heart can block coronary arteries directly, causing acute myocardial infarctions. There is a possibility that such a situation does exist, although it is relatively rare. In the literature, several cases of myocardial infarction associated with metastatic lung cancer have been reported [14,15,16]. As the patient we describe here did not have a diagnosed metastatic disease, we may suspect that COVID-19 and the accumulation of the patient’s other traditional risk factors (smoking, diabetes type 2, hyperlipidaemia, and hypertension) could have contributed to the MI. As a final point, we would like to discuss one more important clinical issue. The treatment of HF involves many different approaches. In 2021, sodium glucose cotransporter 2 (SGLT-2) inhibitors were introduced by European Society of Cardiology guidelines for the treatment of HF with reduced EF as class I agents due to breakthrough studies documenting their efficacy in the reduction of hospitalization rates and mortality [17,18]. However, few studies have been conducted on the effectiveness of SGLT2-inhibitors in HF concerning cancer sub-populations [19,20]. In our opinion, in the reported patient case, the addition of dapagliflozin to standard HF therapy might potentially contribute to the prevention of future worsening episodes of HF. Moreover, it allowed the patient to proceed with chemotherapy, requiring intensive fluid therapy to counteract the potential risk of cisplatin-related nephrotoxicity in a patient with past renal cancer history. The clinical scenario in our case indicates the importance of multidisciplinary team discussions. This was also addressed by recent cardio-oncology guidelines [20]. The dialogue between oncologists and cardiologists for complex patients with cancer and with multiple comorbidities improves the results of diagnostic and therapeutic processes. In particular, a multidisciplinary team approach is necessary when deciding upon starting/continuation vs. interruption of oncologic therapy and in case of potential cancer-therapy-related cardiovascular toxicity development [20].

We introduce this vignette to document how COVID-19 being prolific in the last few years might have not only contributed to and provoked serious adverse events but might have also paradoxically, with the help of modern diagnostic and therapeutic options, led to this patient’s life being saved in the end.

## 4. Conclusions

Cancer and cardiovascular diseases frequently coexist, meaning that an oncological patient is also often a cardiological patient. Diagnostics and cardiological treatment must be carried out simultaneously with anticancer therapies, frequently requiring multidisciplinary team decisions. In order to reduce long-term cardiovascular complications and to prevent the potential discontinuation of cancer therapy due to deterioration of the cardiovascular system condition, early and periodic assessment of the risk of cardiotoxicity and optimal pharmacotherapy are essential.

## Figures and Tables

**Table 1:** Laboratory findings upon patient admission to the hemodynamic lab with myocardial infarction (I) and during hospitalization at the cardiology department due to acute heart failure decompensation (II).

Laboratory Test Results	I	II	Normal Range
C-reactive protein (CRP) [mg/dL]	2.51	7.21	0–0.5
D-dimers [ng/mL]	648	4228	0–500
Troponin T [ng/L]	628.5	14	0–14
Hemoglobin [g/dL]	15.2	15.1	13.7–175
N-terminal prohormone of b-type natriuretic peptide (NT-proBNP) [pg/mL]	2492.6	8163.1	0–125
Aspartate transaminase (AST) [U/L]	64	36	5–34
Blood glucose [mg/dL]	270	207	70–99
Glycated haemoglobin [%]	11.2	10.8	4.8–5.9

## Data Availability

The data are not publicly available due to the nature of the article (case report).
